# Engineering of Three-Finger Fold Toxins Creates Ligands with Original Pharmacological Profiles for Muscarinic and Adrenergic Receptors

**DOI:** 10.1371/journal.pone.0039166

**Published:** 2012-06-14

**Authors:** Carole Fruchart-Gaillard, Gilles Mourier, Guillaume Blanchet, Laura Vera, Nicolas Gilles, Renée Ménez, Elodie Marcon, Enrico A. Stura, Denis Servent

**Affiliations:** 1 CEA, iBiTecS, Service d’Ingénierie Moléculaire des Protéines, Laboratoire de Toxinologie Moléculaire et Biotechnologies, Gif-sur-Yvette, France; 2 Université Pierre et Marie Curie, Paris, France; Universidad Nacional Autonoma de Mexico, Instituto de Biotecnologia, Mexico

## Abstract

Protein engineering approaches are often a combination of rational design and directed evolution using display technologies. Here, we test “loop grafting,” a rational design method, on three-finger fold proteins. These small reticulated proteins have exceptional affinity and specificity for their diverse molecular targets, display protease-resistance, and are highly stable and poorly immunogenic. The wealth of structural knowledge makes them good candidates for protein engineering of new functionality. Our goal is to enhance the efficacy of these mini-proteins by modifying their pharmacological properties in order to extend their use in imaging, diagnostics and therapeutic applications. Using the interaction of three-finger fold toxins with muscarinic and adrenergic receptors as a model, chimeric toxins have been engineered by substituting loops on toxin MT7 by those from toxin MT1. The pharmacological impact of these grafts was examined using binding experiments on muscarinic receptors M1 and M4 and on the α_1A_-adrenoceptor. Some of the designed chimeric proteins have impressive gain of function on certain receptor subtypes achieving an original selectivity profile with high affinity for muscarinic receptor M1 and α_1A_-adrenoceptor. Structure-function analysis supported by crystallographic data for MT1 and two chimeras permits a molecular based interpretation of these gains and details the merits of this protein engineering technique. The results obtained shed light on how loop permutation can be used to design new three-finger proteins with original pharmacological profiles.

## Introduction

Protein engineering can draw inspiration from architectural motifs that have been selected and used in nature to support a large diversity of biological functions. Peptide toxins from venomous animals could be part of the answer. Indeed, although toxins exert their neurotoxic, cardiotoxic or cytotoxic effect by interacting with a large diversity of molecular targets, the number of protein templates selected during the course of evolution to accomplish such vast range of physiological effects appears to be rather limited [1], [2], [3]. The three-finger toxin (3FT) family is widely represented as structural motif, mainly in the *Elapidae* snake venoms, supporting various biological functions [4], [5]. This fold is characterized by three adjacent loops rich in β-pleated sheets emerging from a small globular core containing four invariant disulfide bridges [6]. Despite the small number of residues in the 3FT motif, their targets are numerous and include: L-type calcium channels, integrin receptors, cell membrane phospholipids, acetylcholinesterase, nicotinic acetylcholine or muscarinic and adrenergic receptors [5], making this template attractive in protein engineering. Functional studies by site-directed mutagenesis have identified the sites by which some of these toxins interact with their respective targets. Examples are neurotoxins that interact with muscular and/or neuronal subtypes of nicotinic acetylcholine receptors [7], [8], [9] and with acetylcholinesterase [10]. Transfer of small portions of sequence between an acetylcholinesterase inhibitory toxin to a curaremimetic toxin has resulted in a committal transfer of function [11]. The structure of the modified toxin shows a replication of the loop conformation of the parent [12]. Similarly, grafting the extremity of the central loop of a neuronal nicotinic toxin on the scaffold of a muscular-type toxin confers to it an improved neuronal activity [13]. More recently, using a cDNA display strategy to produce a large peptide library, Naimuddin and coworkers engineered a 3FT by randomizing residues in the three loops in order to obtain modulators of interleukin-6 receptor [14]. All these results emphasize the structural and functional adaptability of the three-finger fold and the ease with which it can support protein engineering.

Muscarinic toxins (MTs) were isolated and purified from the venom of African mambas more than 20-years ago [15], [16] while the identification and purification of adrenergic toxins from mamba or cobra venom occurred more recently [17], [18], [19]. Despite high primary structure identity, often greater than 55%, muscarinic and adrenergic toxins exhibit distinct pharmacological profiles and clear functional differences [17], [18], [20], [21], [22], [23]. For example, MT7 acts as a highly potent and selective antagonist of the M1 receptor subtype [24], [25], [26], [27], [28], through a very stable interaction with the allosteric binding site on the M1 receptor [29]. On the other hand, the mode of interaction of MT1 with muscarinic receptors is less precisely defined, but its selectivity profile for M1 and M4 receptors has been clearly established (review in [20], [21]). Based on previous data reporting adrenergic effects of MT1 in tissue preparations [30], the ability of various MTs to interact with adrenoceptors was investigated, showing that MT1 interacts efficiently with mAChRs and also with α_1A_- and α_2B_-adrenoceptors [23].

**Figure 1:**
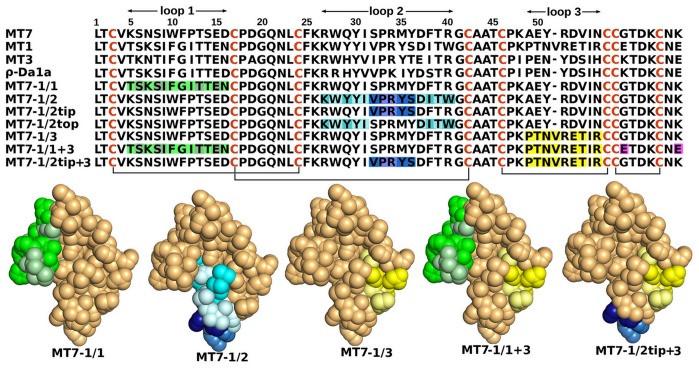
Sequence alignment of the toxins MT7, MT1, MT3 and ρ-Da1a compared to the various chimeras. The grafted residues of MT1 loop 1 are shown in green with conserved residues in a lighter shade generating the chimera of MT7-1/1. For MT7-1/2, MT7-1/2tip and MT7-1/2top the grafted residues of MT1 loop 2 are shown in cyan (top) and blue (tip) and the conserved residues in a lighter shade. For MT7-1/3, MT7-1/1+3 and MT7-1/2tip+3 the grafted residues of MT1 loop 3 are shown in yellow with the conserved residues in a lighter shade. The two extra mutations on the C-terminal section of MT7-1/1+3 that correspond to residues from MT1 are shown in magenta. Below the space-filling representation on the MT7 structure show the positioning of the grafted loops from MT1.

The 23 sequence variations between MT7 and MT1 correspond to residues equally distributed on the three loops of these toxins (Fig. 1). “Loop grafting” has been used to construct seven chimeric toxins to dissect how the affinity, selectivity and functionality for the muscarinic and adrenergic receptors depend on shuffling loops between various toxins. Three chimeras were obtained by substituting loops of the MT7 toxin with the corresponding loop from the MT1. These chimeras were named MT7-1/1, MT7-1/2 and MT7-1/3, numbered for the respective grafted MT1 loop. Given that grafts of the entire loop 2 had enormous effects, this loop was separated into two distinct regions. Two other chimeras were synthesized containing three modifications at the tip of loop 2 (MT7-1/2 tip) or four modifications at the top (MT7-1/2 top), respectively. For sake of completion, two further chimeras were synthesized. These were designed by combining MT7-1/2 tip and MT7-1/3 (MT7-1/2 tip+3) or MT7-1/1 and MT7-1/3 with two additional point mutations found in the C-terminal portion of the MT1 sequence (MT7-1/1+3; Fig. 1). The new designed toxins exhibit original selectivity profiles.

The crystallographic structures of wild-type MT1 and of the engineered chimeric toxins obtained in this study show that despite local structural deviations, the overall integrity of the three-finger fold structure is conserved and the intra-loop hydrogen-bonding maintained. Binding experiments of the natural and engineered toxins on different GPCRs reveal a significant gain in function on α_1A_-adrenoceptor and hM4 muscarinic receptor. It cannot be excluded that these results may have a broader general relevance on a wider spectrum of GPCRs. This engineering study has created novel ligands by combining pre-existing loops. Given the lack of GPCR selective ligands, this approach offers an additional strategy to fill this gap.

## Results

### Synthesis and Folding of Wild-Type and Modified Toxins

The muscarinic toxins, MT7 and MT1, have been synthesised by the one step Fmoc solid phase peptide synthesis approach previously described [25]. We have solved the problems previously reported during the synthesis of the MT1 toxin consisting of failures in the deprotection monitoring and lower couplings because of local chain aggregation. These occurred in two regions; the central part of the sequence (Val-32 - Tyr-35) and the N-terminal end (Leu-1 - Ser-8). The solution was to replace Thr-13 and Thr-39, two residues introduced during the synthetic process before the two critical regions, by a pseudo-proline residue. We therefore incorporated the dimethyloxazolidine of the di-peptide Ile-Thr at positions 12–13 and 38–39 during the synthesis. This chemical modification disrupts peptide chain aggregation in the same manner as a proline does [31]. We took advantage of this improvement during the synthesis of the different chimeric toxins. The synthesis of MT7-1/2 top and MT7-1/3 were undertaken in a similar manner as that described for MT7 using Fmoc amino acids and the efficient coupling reagents HOAT or CL-HOBT/DCCI. However, for the other chimeric toxins, the dipeptide Ile-Thr was introduced using Fmoc-Ileu-Thr (Ψ^MeMe^pro)-OH at positions 12–13 in the MT7-1/1 or MT7-1/1+3 and 38–39 in MT7-1/2, MT7-1/2 tip and MT7-1/2 tip+3. A longer coupling time was used for this reaction. During the synthesis of the different toxins no failure in the deprotection monitoring was observed. At the end of the synthesis, regeneration of the Thr from the oxazolidine occurs during the course of the normal TFA-mediated cleavage reaction and each toxin yielded a crude mixture in which the main component corresponds to the reduced form of each chimera toxin.

Folding of the different chimera toxins was achieved using the experimental conditions that were successfully applied to the MT7 and MT1 toxins. An efficient and rapid folding process was followed for MT7-1/1 and MT7-1/3 but not for MT7-1/2, MT7-1/2tip and MT7-1/2top. To find out a common optimal refolding condition, we screened different additives and various concentrations of reduced and oxidized glutathione. The final optimized refolding buffer containing 30% glycerol, 0.1 M Tris-HCl, 1 mM EDTA, 1 mM GSH, 1 mM GSSG, pH 8 was used to prepare large amounts of the different chimeras (refolding yield; 20–30%). Amino acid composition and electro-spray mass analysis confirmed the purity and identity of the different proteins. The masses were 7359.2 for MT7-1/1 (theoretical 7359.4), 7538.5 for MT7-1/1+3 (theoretical 7538.6), 7443.3 for MT7-1/2 (theoretical 7443.5), 7440.2 for MT7-1/2tip (theoretical 7440.5), 7546.5 for MT7-1/2tip+3 (theoretical 7546.6) 7475.2 for MT7-1/2top (theoretical 7475.6) and, 7578.7 for MT7-1/3 (theoretical 7578.8).

The CD spectrum analysis of the MT7, MT1 and chimeric toxins reveals a typical β-sheet signature in these toxins with the maximum and minimum signals observed at 196 and 210 nm, respectively (see Figure S1, Supporting Information). An additional distinct signature at 230 nm, previously reported for MT1 [25] and characteristic of the two substitutions Q29Y and R40W, is also observed for MT7-1/2 and MT7-1/2 top. A similar effect was also observed for erabutoxin, due to an aromatic residue at position 29 [32].

### Crystallization and Crystallographic Structure Determination

**Figure 2:**
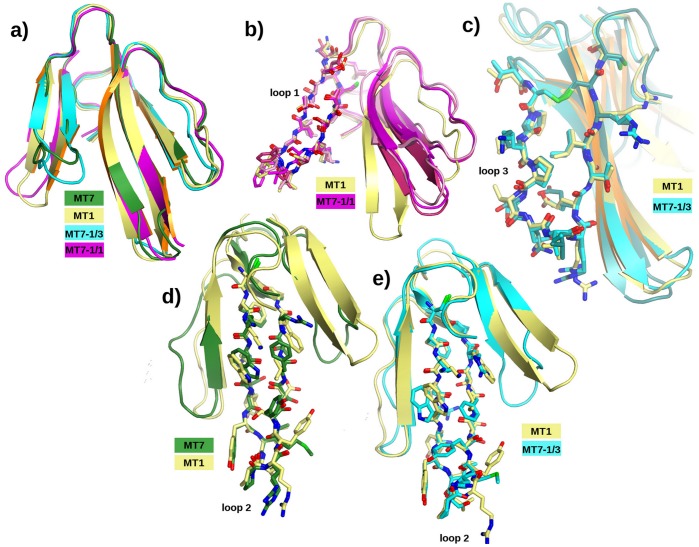
Schematic representation of MT7 (green), MT1 (gold) and chimeras MT7-1/1 (magenta) and MT7-1/3 (cyan). **a)** Toxins superimposed globally on each other. The largest deviations are observed on loop 1 and the tips of the various loops. **b)** Chimera MT7-1/1 superimposed on MT1 on loop 1 shown in stick representation. **c)** Chimera MT7-1/3 superimposed on MT1 on loop 3 showing that although loop 2 remains entirely that of MT7 and that there are strong interactions between loop 2 and loop 3, loop 3 maintains strictly its conformation as on MT1. **d)** As shown by the superimpositions of loop 2 from MT1 and MT7, although the backbone matches well, to maintain the interaction between loop 2 and loop 3 and the conformation of loop 3 as in MT1, the side chain orientations on loop 2 of MT7 require adaptive changes. **e)** The side chain orientations of loop 2 of chimera MT7-1/3 resemble those of loop 2 of MT1 although the sequence is that of MT7.

Crystals of MT1 and MT7-1/1 toxins were obtained by sitting drop vapor diffusion and MT7-1/3 by slow evaporation close to 48 mg/ml protein concentration. The crystallographic structures of the MT7-1/1 and MT7-1/3 toxins were solved by molecular replacement with the MT7 diiodo-Tyr51 (PDB code = 2VLW) [28] as a starting model while MT1 was solved using toxin ρ-Da1a (unpublished) as starting model. The structures were refined to 1.3 Å, 1.39 Å and 1.8 Å resolution, respectively (Table S1). The asymmetric unit of MT1 (PDB code  = 4DO8) is composed of two toxins, an acetate and a thiocyanate ions, the latter ion, positioned between the two molecules in the asymmetric unit, might be important for the crystallization. The two molecules are virtually identical and superimpose with a mean deviation of 0.32 Å (rmsd  = 0.74 Å) on all atoms and an rmsd 0.15 Å on 256 main chain atoms. The asymmetric unit of chimera MT7-1/1 (PDB code  = 3FEV) consists of three toxins that superimpose well on each other but with larger deviations compared to MT1 (rmsd between chains A/B  = 0.88 Å; A/C  = 0.73 Å; B/C  = 1.10 Å on all main chain atoms). The major variations are at the tips of loops 2 and 3, the region that was not manipulated. MT7-1/3 (PDB code  = 3NEQ) counts two molecules in the asymmetric unit with an rmsd of 1.47 Å between the two molecules on all 207 main chain atoms. The superimposition of the different copies of each toxin infers that these structures are uniquely defined while retaining a certain degree of flexibility. The canonical three-finger fold structure comprising five β-strands forming a twisted β-sheet is strictly abided to (Fig. 2a).

### Structure Comparisons

The structures of the MT7 diiodo-Tyr51 (PDB code = 2VLW), MT1, MT7-1/1 and MT7-1/3 described here, were superimposed with COOT [33] and pyMOL to understand the structural effect of loop grafting on the individual loops (Fig. 2) and on the global superimposition of the various structures (see Figure S2, Supporting Information). The loops with the MT1 sequence grafted onto the MT7 scaffold superimpose well on those found on the parent toxin (Fig. 2). Loop 1 of each of the three molecules in the asymmetric unit of MT7-1/1 superimposes precisely on that of loop 1 of MT1 (Fig. 2b). The loop separates from loops 2 and 3 as it does in MT1 but to a greater extent. The comparison of loop 3 from each of the two molecules in the asymmetric unit of MT7-1/3 compared to MT1 shows again an excellent match (Fig. 2c). The comparisons of loop 2 from MT1 and MT7 shows that the backbone of this loop is conserved between the two natural toxins, but the side chains adopt a different orientation (Fig. 2d). The comparison of loop 2 from chain A of MT7-1/3 with loop 2 of MT1 shows that the replacement of loop 3 of MT7 for that of MT1 imposes a reorientation of the side chains to match that of MT1 although there is poor sequence identity (Fig. 2e).

MT1 superimposition onto MT7 by least-squares fit shows large deviations in both the loops and in the sequence-conserved disulphide-bridged scaffold leading to important differences when the main chain atoms are compared (rmsd MT1 vs MT7 = 3.22±0.01 Å, depending on the molecule in the asymmetric unit that are compared). A better match can be obtained by not matching consecutive residues but only the secondary-structure elements. Secondary-structure matching (SSM) [34] for the MT1/MT7 comparison leads to a value of around 1.34 Å where the matched residues have a reduced sequence identity of only 66%. Given that loop 3 of MT1 is longer than that of MT7, the least-squares fit method is also inappropriate. In supplementary figure S2 we use the relatively good conservation of the conformation of loop 2 and its interaction with the second strand of loop 3 to guide the overall structural superposition. This allows for a visual rather than mathematical scoring of the alignment. To better understand the comparison by the two methods we can consider the superposition of MT1 with chimera MT7-1/3 that counts the same number of residues (Fig. 1). By least-squares fit we have an rmsd for MT1 vs MT7-1/3 of 2.87±0.08 Å while by SSM the value falls to an rmsd of 0.89 Å on 61 aligned residues with a 79% sequence identity. The superimposition obtained by matching only loop 2 and the second strand of loop 3 agrees better with the SSM value (see Figure S2, Supporting Information). Comparing by least-squares fit MT1 to chimera MT7-1/1 is less reliable because of a difference in sequence length which gives an rmsd between MT1 and MT7-1/1 of 2.38±0.03 Å. By SSM the rmsd remains high at 1.64 Å. This is mainly because the algorithm aligns 64 residues instead of only 61 in the previous comparison where 3 extra residues on loop 1 were omitted due to excessive deviation. If MT7 is compared to chimera MT7-1/1, least-squares gives an rmsd of 3.13±0.02 Å while by SSM gives 1.4 Å. In this comparison SSM uses only 59 residues. The SSM superimposition for MT7/MT7-1/3 gives an rsmd value of 2.8 Å with only 47 residues matched with a 91.5% identity, in good agreement with the SSM values (see Figure S2, Supporting Information).

### Equilibrium Binding Studies of Wild-Type and Chimeric Toxins to the hM1, hM4 Muscarinic Receptors and α_1A_-Adrenoceptor

**Figure 3:**
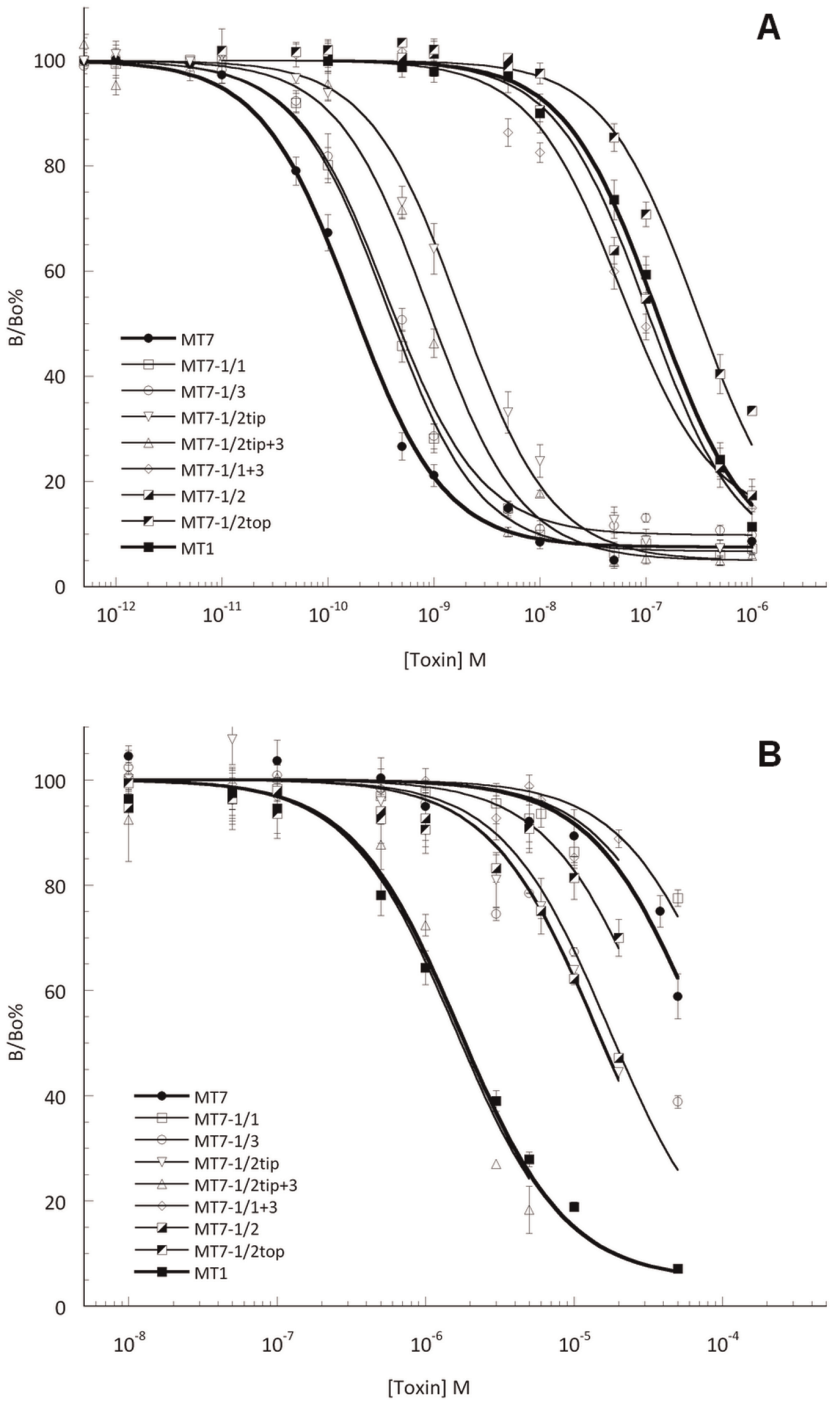
Inhibition of [^3^H]-NMS binding to hM1 and hM4 receptors by wild-type MT7, MT1 and chimera toxins. Binding experiments were performed by incubating hM1 (A) or hM4 (B) membrane fractions of receptor with [^3^H]-NMS (0.5 nM) and varying concentrations of toxin at room temperature and overnight. The total specific binding in each experiment was 1500±300 cpm. The results are expressed as the ratio of the specific [^3^H]-NMS binding measured with (B) or without toxin (B_o_). All experiments were performed at least three times in duplicate.

MT7 and MT1 toxins interact with muscarinic and adrenergic receptors with an affinity of 34 pM and 24 nM for M1, 13 µM and 310 nM for M4 and greater than 20 µM and 62 nM for α_1A_, respectively. To identify the 3FT regions responsible for these large differences in affinity which could be submitted to engineering, we analyzed the impact of substitutions in each loop of MT7 on its binding affinity on the different receptor subtypes. The capacity of wild-type and chimeric toxins to inhibit the binding of [^3^H]-NMS to hM1 or hM4 membrane preparations was measured (Fig. 3a, 3b), allowing the calculation of their pK_i_ (Table 1). Substitutions within loops 1 and 3 weakly affect the toxin’s interaction with the hM1 receptor, corresponding to a 2-fold decrease in affinity. In contrast, grafting the entire loop 2 of MT1 into the MT7 scaffold (MT71/2) induces a drastic 400-fold drop in affinity for the M1 receptor (Table 1). Interestingly, a similar effect was observed with the MT7-1/2 top chimera with only four modifications: R27K, Q29Y, F38I, and R40W. Changing the tip of the central loop (MT71/2 tip chimera) affects moderately toxin binding with a relatively low 7-fold decrease in affinity. Moreover, combining these later substitutions (S32V, M35Y and Y36S) with the grafting of loop 3, leading to the MT7-1/2 tip+3 chimera, confirms the low impact of the modifications introduced in these two regions of the toxin for the hM1 interaction. Finally, combining loops 1 and 3 substitutions with two additional modifications in the C-terminal part (G59E, K65E) (MT7-1/1+3) leads to a severe 360-fold decrease in affinity for the hM1 receptor (Table 1).

**Table 1:** Affinity constants of wild-type MT7, MT1 and chimeric toxins for M1, M4 muscarinic receptors and α_1A_-adrenoceptor.

	M1		M4		α_1A_	
	pKi	Ki tox/Ki MT7 loss of function	pKi	Ki MT7/Ki tox gain of function	pKi	Ki MT7/Ki tox.gain of function
**MT7**	10.47±0.04	1	4.87±0.06	1	>4.7	1
**MT7-1/1**	10.16±0.05	2	>4.7	<0.67	7.10±0.11	>252
**MT7-1/2**	7.86±0.02	406	5.59±0.04	5.3	>4.7	1
**MT7-1/3**	10.04±0.11	2.6	5.51±0.08	4.4	6.97±0.09	>187
**MT7-1/2tip**	9.57±0.13	7.5	5.60±0.08	5.4	>4.7	1
**MT7-1/2top**	7.62±0.07	706	>4.7	<0.67	>4.7	1
**MT7-1/2tip+3**	9.83±0.02	4.4	6.58±0.01	52	6.36±0.01	>46
**MT7-1/1+3**	7.91±0.02	362	>4.7	<0.67	8.47±0.08	>5900
**MT1**	7.62±0.04	706	6.50±0.04	43	7.21±0.16	>325

On the hM4 receptor, only MT1 was able to displace completely the radiotracer in the range of concentrations used (Fig. 3b). Nevertheless, the incomplete inhibition curves obtained for all the other toxins suffice to give a good approximation of their affinity constants (Table 1). The affinity of MT7 for hM4 is very low (pKi: 4.87±0.06), approximately 40-fold less than that of MT1 (pKi: 6.50±0.04). Modifications introduced in the MT7-1/1, MT7-1/1+3 and MT7-1/2 top chimeras affected only weakly the affinity for the hM4 receptor (less than 2-fold decrease), whereas the MT7-1/2, MT7-1/2 tip and MT7-1/3 increase the affinity by 4- to 5-fold. It is worth noting that the gain in affinity obtained by combining MT7-1/2 tip and MT7-1/3, in the MT7-1/2 tip+3 chimera, is additive achieving an overall 50-fold increase for the hM4 receptor (Table 1).

**Figure 4:**
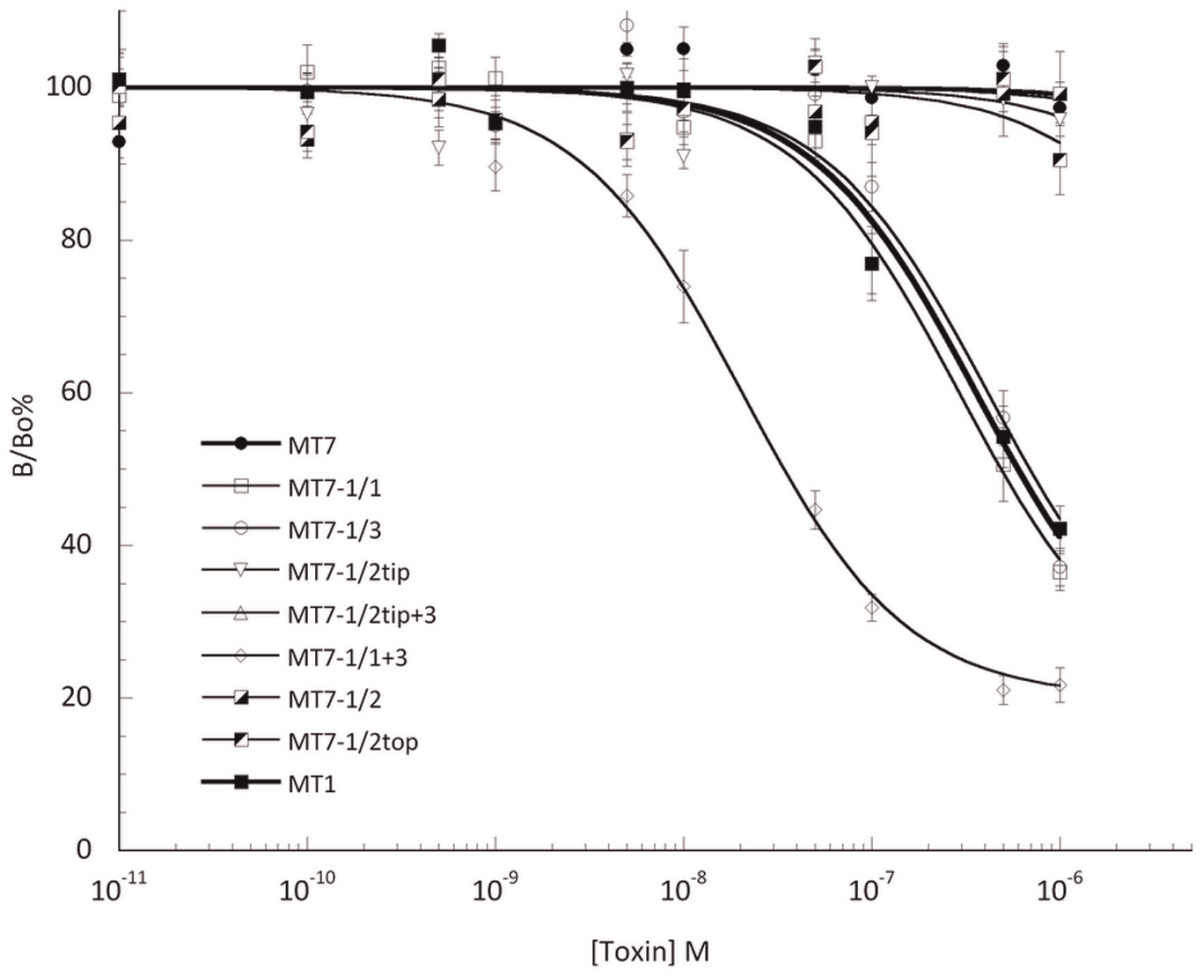
Inhibition of [^3^H]-Prazosin binding to α_1A_-adrenoceptor by MT7, MT1 and chimeric toxins. Binding experiments were performed by incubating α_1A_-AR membrane fractions of receptor with [^3^H]-Prazosin (1.5 nM) and varying concentrations of toxin at room temperature and overnight. The results are expressed as the ratio of the specific [^3^H]-Prazosin binding measured with (B) or without toxin (B_o_). All experiments were performed at least three times in duplicate.

Equilibrium binding experiments with [3H]-Prazosin show that MT1 and MT7 toxins interact with α_1A_-adrenoceptor with relatively high (pKi: 7.21±0.16) and very low affinity (pKi >4.7), respectively. A 200-fold increase in affinity was obtained by the independent grafting of either loop 1 or 3, enough to elevate the affinity of MT7 to the level of MT1 (Table 1; Fig. 4). The partial or total insertion of the loop 2 was ineffective. Remarkably, a 6000-fold increase in affinity compared to MT7 was achieved on the α_1A_-adrenoceptor by grafting a combination of loops 1 and 3 (MT7-1/1+3; pKi: 8.47±0.08), corresponding to a 20-times affinity increase relative to MT1 (Table 1).

## Discussion

Because they are resistant to degradation, permissive to mutations and tolerant to large insertions or deletions, three-finger fold toxins (3FT) have been largely exploited by snakes to support various toxic functions in their venoms [5]. These characteristics make the three-finger fold template suitable for protein engineering [11], [13], [14]. Structural knowledge to drive the 3FT engineering is still lacking in the context of 3FT-GPCRs complexes although molecular dynamics simulations have identified regions susceptible to large-scale motions that could be permissive to modification [35]. Using the interaction of 3FT toxins with muscarinic or adrenergic receptors as a model system, we have addressed this question by a loop grafting strategy producing seven chimeric toxins by chemical synthesis. These chimeras are the results of the transfer of MT1 loops into the MT7 template (Fig. 1).

The structural impact of loop grafting was analyzed by X-ray crystallography. The three-dimensional structures of chimeras MT7-1/1 and MT7/1-3 were determined and compared to those of the natural toxins MT1 and MT7, previously reported [28].?The comparison shows which features are transferred from parent to chimera and which features are more variable and could give rise to the new functionality. Given the high resolution of the structures and the presence of multiple copies of each toxin in the crystal asymmetric unit, it leaves no doubt that the amino acid sequence determines the conformation of the individual loops and that the loop conformation is transferred together with the loop. The structures provide irrefutable evidence that for loops 1 and 3 grafted from MT1 onto the scaffold of MT7 retain their original conformation (Fig. 2a, 2b). Another invariable is that the backbone conformation of loop 2 is well conserved between MT7 and MT1, although the sequence is not. Loop grafting preserves the overall three-finger structure in all chimeric toxins. The spatial relationship of the grafted loops with the remaining part of the structure is variable. The grafted loop 1 of chimera MT7-1/1 preserves its structure as in MT1 but does not adopt the same relationship with loop 2 as in MT1 nor keep the relationship found between loop 1 and loop 2 of MT7. Thus a new toxin with novel properties is created by the grafting. The situation with loop 3 is different because of the conserved interaction between loop 2 and the second strand of loop 3. To preserve the interaction we have observed that the side chain orientations on the top part of loop 2 tend to conform to those from the parent toxin. Indeed, in chimera MT7-1/3 the side chains on loop 2 execute a concerted rotation and adopt conformers that match closely those on MT1 loop 2. This observation is linked to the change from Arg-40 at the top of loop 2 of MT7 to Trp in MT1. The flexible Arg side chain can adopt the same side chain orientation as the Trp (Fig. 2e) and because such orientation is not compatible with that of the other side chains on the loop, the side chains are forced to change. The effect that this could have on receptor binding is unpredictable. These movements are important in explaining the effect of grafting of loop 2, in particular the top of the loop. The tip of this loop is relatively independent of the rest of the toxin in MT7 but in MT1 with the longer loop 3 this is no longer the case (see Figure S3, Supporting Information). This is important when comparing toxins with different lengths of loop 3 (Fig. 1).

Since GPCRs are among the targeted receptors for the three-finger fold class of toxins, it can be envisaged that the scaffold forming the basis of their diversity could be engineered to yield novel ligands, some of which would be selective for GPCRs not yet targeted in nature. To evaluate this hypothesis, we have approached the process of diversification by the “loop grafting” strategy and we have produced 3FT chimeric toxins with new pharmacological profiles towards muscarinic and adrenergic receptors, with affinities that sometimes surpass those of the two parent toxins.

Grafting the entire MT1 loop 2 (chimera MT7-1/2) onto the MT7 template provokes a large decrease in affinity of the toxin for hM1 receptor (400-fold), a weak increase for hM4 (5-fold) and no effect on α_1A_-adrenoceptor (Table 1). Comparison of the affinities of the MT7-1/2top and MT7-1/2tip chimeras on the three receptors with those of the MT7-1/2 toxin confirms the lack of effect of the modifications introduced in this region on the α_1A_-adrenoceptor binding and suggests that the drastic loss in function on hM1 and the moderate gain in function on hM4 are mainly associated with the top and the tip of loop 2, respectively. Indeed, with only four substitutions compared to MT7 (R27K, Q29Y, F38I and R40W), MT7-1/2top reaches an affinity for hM1 equal to that of MT1 while all chimeras have a higher affinity. Of these four substitutions, the F38I mutation involves a 2-fold loss in affinity [36] and the R27K substitution is relatively conservative, suggesting that the MT7-specific residues, Gln-29 and Arg-40 might play an important role in the hM1 receptor interaction. The R40W is important for the relationship between loop 2 and loop 1 (Fig. 2e, see Figure S3, Supporting Information). Nevertheless, previous results using punctual alanine modification have shown the crucial role of residues at the tip of loop 2, such as Arg-34 and Tyr 30, in the MT7-hM1 interaction [25], [29]. Conservation of these residues in MT7-1/2tip chimera may explain the relative high affinity of this toxin and suggests that the three substitutions included in this chimera (S32V, M35Y, Y36S) induced the moderate affinity decrease for hM1 receptor (7-fold). Similarly, these substitutions play a positive role in the hM4 recognition, as shown by the 5-fold increase in affinity observed on this receptor (Table 1). Thus, loop 2 is highly critical in the picomolar affinity of the MT7-hM1 interaction, weakly involved in the hM4 recognition and plays no role in the α_1A_-adrenoceptor binding.

On hM1 receptor, substitutions of MT7 loops 1 and 3 by those of MT1 (chimeras MT7-1/1 and MT7-1/3) have no significant impact on the toxin affinity (Table 1), suggesting that the seven modifications associated with each of these substitutions were not involved in the high affinity of the MT7-hM1 interaction. Even if these results do not exclude a complementary role of the toxin loops 1 and 3 in the MT7-hM1 receptor interaction, as proposed on the structural model of this complex [29], they are consistent with previous results obtained with punctual alanine modifications, reporting that some modifications in loops 1 and 3 of MT7 have weak or no effect on the toxin’s affinity for the hM1 receptor [28]. While loop 1 transfer has no significant effect on the toxin affinity for hM4, a moderate gain of function (5-fold) is observed with the grafting of loop 3 (Table 1). Notably, a two orders of magnitude increase in affinity for the α_1A_-adrenoceptor is achieved for chimeras MT7-1/1 and MT7-1/3 by the grafting of loop 1 or 3. These toxins are characterized by an affinity close to that of MT1 for the α_1A_-adrenoceptor.

Combining some of the previous grafting in an attempt to optimize our engineering, leads to new chimeric toxins with affinities which surpasses that both of natural parents. For example, the weak gain in function on hM4 receptor consecutive to the loop 3 or loop 2tip substitutions is largely enhanced on the MT7-1/2tip+3 which combines both modifications. Thus, a 50-fold gain in function is observed with this chimera (Table 1). In nature, the MT3 toxin has evolved to reach an affinity of 2 nM for the hM4 receptor [23], [37]. Comparing its sequence with those of MT7, MT1, MT7-1/2tip+3 (Fig. 1) reveals that the chimera comes close to matching the MT3 sequence. The most interesting changes are localized at the tip of loops 2 and 3. These are Val_MT1_/Ile_MT3_/Ser_MT7_-32, Tyr_MT1&MT3_/Met_MT7_-35, Ser_MT1_/Thr_MT3_/Tyr_MT7_-36 at the tip of the loop 2 and Pro_MT1&MT3_/Ala_MT7_-49, Asn_MT1&MT3_/Tyr_MT7_-51, Thr_MT1_-55/Ser_MT3_-54/Val_MT7_-54 and Arg_MT1_-57/His_MT3_-56/Asn_MT7_-56 at the tip of the loop 3. The MT7 specific residues, Ser-32, Met-35, Tyr-36, Ala-49, Tyr-51, Val-54 and Asn-56 are likely to be responsible for the exceptional selectivity of this toxin for hM1 relative to the hM4. Loop 3 would appear to be responsible for the receptor subtype selectivity. More strikingly, the simultaneous grafting of the loops 1 and 3 of MT1 on the MT7 scaffold provokes on α_1A_-adrenoceptor a cumulative effect as compared to those obtained with separate permutations. Thus, the MT7-1/1+3 chimera reaches an affinity which is 6000 times greater that MT7 and even 20 times more potent than MT1 on this receptor subtype (3.4 nM; Table 1). The major role of the loop 1 in this interaction is coherent with the large sequence identity (80–90%) in this region between MT1 and the two most potent toxins that target this receptor subtype: MT3 (1.6 nM) and ρ-Da1a (0.3 nM) (Fig. 1) [17], [23]. Since loop 3 of MT3 and ρ-Da1a are identical, the differences in their affinity for α_1A_-adrenoceptor are probably due to sequence variations on loop 1. It is interesting to note that the chimera MT7-1/1+3 is built by loops 1 and 3 of MT1 grafted simultaneously on the MT7 skeleton with the additional G59E-K65E modifications present on MT1 (Fig. 1, see Figure S3, Supporting Information). This chimera is MT1-like with only the central loop of MT7 grafted onto the MT1 template and displays an affinity for hM1 receptor twice that of MT1. The transposition of loop 2 from MT1 to MT7 (chimera MT7-1/2) or from MT7 to MT1 (chimera MT7-1/1+3) leads to the same affinity on hM1. This corresponds to a 2-fold gain compared to MT1 but a 400-fold loss compared to MT7. This suggests first that the two Glu insertions and more particularly the C-terminal charge inversion would be involved in the decrease in affinity of the MT7-hM1 interaction and secondly that the relationship between the loops and not just loop 2 is important in the interaction with the hM1 receptor. This result is structurally very important as it highlights how selectivity can be modulated through loop grafting. The MT7-1/1+3 toxin possesses a unique pharmacological profile among all the muscarinic and adrenergic toxins with a high affinity for hM1 and α_1A_ receptors and no interaction with hM4.

In conclusion, the loop transfer engineering methodology described here is an effective means of creating new functionality onto the 3FT scaffold by connecting together a relatively small number of LEGO-like blocks. By this approach, we were able to produce chimeric toxins with affinity towards receptors that surpass that of the two parent toxins, thus creating ligands with unique pharmacological profiles. Since only seven chimeric toxins have been chemically synthesized starting from loops from just two toxins (MT1 and MT7; Fig. 1), the gain in function is very encouraging, considering that the engineering database from which loops could be selected is vast. Furthermore, functional regions that have been identified can later be improved by display methodology. Comparison of the chimeric toxins sequences developed in this study with those of natural toxins like MT3 and ρ-Da1a reveals certain similarities, suggesting that a “loop permutation-like” strategy might happen in nature to diversify the toxins functions to respond to modifications of the snake’s environment. This hypothesis can be correlated with the recently proposed ASSET strategy, suggesting that an accelerated segment switch in exons in 3FT may alter their molecular targets selection by inducing change or gain of functions [38], [39]. Since the three-finger toxin fold was known to interact with diverse targets including various biogenic amine GPCRs, our data are likely to be of broad general relevance.

## Materials and Methods

### Materials

[^3^H]-N-Methylscopolamine ([^3^H]-NMS; 78 Ci/mmol) and 7-Methoxy-[^3^H]-prazosin ([^3^H]-prazosin; 85 Ci/mmol) were obtained from PerkinElmer (Courtaboeuf, France). Atropine, N-methylscopolamine and prazosin were purchased from Sigma-Aldrich (St Quentin-Fallavier, France).

### Peptide Synthesis

Assembly of the different chimeric proteins were performed on a standard Applied Biosystems 433 peptide synthesizer (Applied Biosystems, France) and carried out using the stepwise solid-phase method with dicyclohexyl-carbodiimide/HOAT (1-hydroxy-azabenzotriazole) or 6-Cl-HOBT (6-chloro-1-hydroxybenzotriazole) as coupling reagents and N-methyl pyrrolidone as solvent. Fmoc-protected amino acids were used with the following side-chain protections: t-butyl ester (Glu,Asp), t-butyl ether (Ser, Thr, Tyr), trityl (Cys, His, Asn, Gln), 2,2,5,7,8-pentamethyl-chromane-6-sulfonyl (Arg), t-butyloxy-carbonyl (Trp). The Fmoc-pseudoproline dipeptide, introduced at different positions in the chimera toxins was Fmoc-Ileu-Thr (Ψ^MeMe^pro)-OH. The MT7-MT1- chimera toxins were assembled on a Fmoc-Glu(OtBu)-Wang resin (loading: 0.5 mMol/g) or Fmoc-Lys(Boc)-Wang resin (loading: 0.55 mMol/g). The different syntheses were run on a modified version of the Applied Biosystems standard 0.1 mmole small-scale program using 0.05 mmole of each resin. This program achieves UV monitoring of the deprotection step. When the deprotection is too slow after two and/or three successive deprotections of 3 min, it automatically extends the deprotection time by 20 min and the coupling time (normal coupling: 30 min or 60min in case of pseudoproline dipeptide) by an extra 30 min. After each coupling, the resin was acetylated by a mixture of 5% acetic anhydride, 6% 2,4,6-collidine in DMF. At the end of the synthesis the peptide-resins were treated with trifluoroacetic acid (9 ml), triisopropylsilan (0.5 ml), and 0.5 ml distilled water. The peptides were then cleaved from the resin and the protecting groups were removed from amino acid side-chains. After two hours of incubation, the mixture was filtered in cold tert-butyl methyloxide and centrifuged three times. The precipitates were dissolved in a solution of 10% acetic acid and lyophilized. The toxins were purified by reverse phase HPLC using a Discovery BioWidepore C5 column (Supelco,PA, USA) (250×10 mm) with a gradient of 40 to 60% of solvent B in 40 min, (A: 0.1% TFA in H_2_O, B: 60% acetonitrile and 0.1% TFA in H_2_O) The flow rate was 4 ml/min and the detection was followed at 280 and 214 nm.

### Optimization of Disulfide Bond Formation, Purification and Characterization

The reduced synthetic and chimera toxins were subjected to an oxidative reaction in 0.1 M Tris, 1mM EDTA buffer (pH 7.8) containing 0.5 M guanidine hydrochloride in the presence of reduced (GSH) and oxidized (GSSG) glutathione in a molar ratio of 1/10/100 peptide/GSSG/GSH at a concentration of 0.05 mg/ml as described elsewhere (12) or submitted to a refolding screening procedure. In this case, a reduced-denatured toxin, was screened against 40 different refolding conditions, a combination of 8 buffers (100 mM Tris-Cl, 1 mM EDTA pH 8 with or without 100 mM NaCl, or 30% glycerol or 0,25% triton X100 or 100 mM NDSB-256 or 0.05% PEG3500 (w/v) or 0.5 M Gnd-Cl or 0.5 M Arg) and 5 concentrations of the redox couple of reduced glutathione (GSH) and oxidized glutathione (GSSG): 10/1 mM, 5/1 mM, 1/1 mM, 5/0.5 mM, 1/0.5 mM. To minimize the adsorption of protein on to the vessel’s surface the refolding was carried out in a microsorb eppendorf tube of 1 ml. Each sample was reduced for 2 h at 37°C in 6 M guanidine-HCl, 10 mM dithiothreitol (DTT), 50 mM Tris, pH 8 denaturing buffer. Refolding was initiated by a 1/200 fold rapid dilution in ice cold refolding buffer to a final concentration of 20 µg/ml and a final volume of 1 ml followed by incubation at 4°C for 72 h. Refolding efficiency was evaluated by integrating the surface of the peak corresponding to the refolded form of the protein after analytical HPLC separation. Typically, the pH of each sample was lowered to pH 3 by addition of 30% TFA and 200 µl loaded in an autosampler (Waters 717plus) connected to an HPLC chain (Waters 600) and a photodiode array detector (Waters 996). Fast separation was performed on a Chromolith SpeedRod, RP18e, 50–4.6 mm column (Merck, Darmstadt,Germany) by means of a 10 min linear gradient of acetonitrile in 0,1% (by vol.) trifluoroacetic acid from 25 to 35% at a flow rate of 4 ml/min, detection 214 nm.

Large scale refolding was performed by diluting the reduced protein 200 fold in the optimized refolding buffer namely 100 mM Tris-HCl, 1 mM EDTA, 30% glycerol, 1 mM GSH, 1 mM GSSG pH 8 to a final concentration of 0.1 mg/ml and then incubated at 4°C for 72 h. After acidification the purification of the refolded toxins were performed on a Discovery BioWidepore C5 column (Supelco, PA, USA) (250×10 mm) with a gradient of 40 to 60% of solvent B in 40 min, (A: 0.1% TFA in H_2_O, B: 60% acetonitrile and 0.1% TFA in H_2_O) The flow rate was 4 ml/min and the detection was followed at 280 and 214 nm. Finally, each protein was analytically characterized on analytical reversed-phase HPLC a Discovery BioWidepore C5 column (Supelco,PA, USA) (100×4.5 mm) with a gradient of 40 to 60% of solvent B in 40 min, (A: 0.1% TFA in H_2_O, B: 60% acetonitrile and 0.1% TFA in H_2_O). The flow rate was 1 ml/min and the detection was followed 214 nm. For the amino acid analysis, the hydrolysates obtained after acid hydrolysis in a sealed vial were heated at 120°C in the presence of 6 N HCl for 16 h and analysed using an Applied Biosystems model 130A automatic analyzer equipped with an online 420A derivatizer for the conversion of the free amino acid into phenyl thiocarbamoyl. Mass determination was performed on a Nermag spectrometer coupled to an analytical (Brandford) electrospray source. The concentrations of the different toxins were evaluated spectrometrically.

### Circular Dichroism Analysis

CD spectra were recorded on a Jasco J-815 CD spectrometer. Measurements were routinely performed at 20°C in 0.1 cm path-length quartz cells (Hellma, Paris, France) with a peptide concentration of 10^–5^ M in water. Spectra were recorded in the 186 to 260 nm wavelength range. Each spectrum represents the average of at least three spectra.

### Crystallization

The crystallization of the MT7 wild-type toxin in its diiodo-Tyr51 derivative had been reported previously [28]. Briefly, lyophilized synthetic toxin was re-suspended at 5 mg/ml in 0.02% azide, 50 mM sodium acetate, pH 4.6. The reservoir solution used was 1.25 M ammonium sulfate, 90 mM sodium citrate, and 10% methyl pentanediol (MPD), pH 5.5. Similarly, lyophilized synthetic toxin MT1 was re-suspended at 8.3 mg/ml in 0.02% azide, 50 mM sodium acetate, pH 5.5 and crystallized by sitting drop vapor diffusion over a reservoir consisting of 36% mono-methyl polyethylene glycol 2,000 (MPEG 2K), 450 mM NaCl, 90 mM KSCN, 100 mM imidazole-HCl, pH 7.5. Crystals appeared after 1 month. No cryoprotectant was needed for flash-cooling given the high MPEG concentration used to crystallize the toxin. The chimera MT7-1/1 was prepared from 1.3 mg of lyophilized synthetic toxin dissolved in 50 mM Na acetate, pH 5.5, with 0.02% azide to yield a 5 mg/ml solution. Screening for crystallization was carried out by sitting drop vapor diffusion at 20°C in a cooled incubator using 4 selected conditions from the “Stura” screens [40] (Molecular Dimensions) and the conditions that had yielded crystals for the wild type MT7. Conditions that yielded crystals were further refined. The crystals used for data collection were grown from 1.2 M ammonium sulfate, 90 mM sodium citrate, with 4% MPD, 6% propanol at pH 5.5. The crystals were transferred to a cryo-solution consisting of 1.5 M ammonium sulfate, 75 mM sodium citrate, pH 5.5, 25% glycerol and 5% MPD. After a short solvent exchange step the crystals were cryo-cooled in liquid ethane. Chimera MT7-1/2 was found to be less soluble than MT7-1/1, tended towards filamentous aggregation. A similar problem had been encountered with the first batch of toxin MT1 from which crystals could not be obtained given the limited quantities available insufficient to carry out a more complete screen. Chimeric toxin MT7-1/3 was prepared from 0.48 mg resuspended in 0.02% azide, 50 mM sodium acetate at pH 5.5 to yield a 5 mg/ml solution. Needle crystals of this chimera were obtained from 1.02 M ammonium sulfate, 50 mM sodium citrate, 1% MPD at pH 5.5 using the same conditions as for MT7-1/1 without any need for screening. Better crystals were obtained later by the slow evaporation method over a period of 3 months at 4°C in a laboratory refrigerator. The method consists in allowing moisture to escape slowly over a long period of time. This can be done by loosening the cap of an eppendorf tube containing the protein. However, we used a more reproducible method that consists in replacing the screw cap by one from a different manufacturer and screwing in the cap tightly. Because the specifications of the two manufacturers differ, this still allows water vapor to leak out at a rate that is compatible with crystallization while it is difficult to evaluate the rate of vapor escape from a loosened cap. When the crystals were harvested, the effective concentration of the protein was estimated from the decrease in sample volume to be 48 mg/ml. The crystals were stabilized in two steps: first in a solution consisting of 0.96–1.26 M ammonium sulfate, 65 mM sodium citrate with 5% MPD, pH 5.5 over a period of at least one day and then in 80% saturated lithium sulfate before cryo-cooling in liquid ethane.

### Data Collection and Crystal Structure Determination

Data for the all the crystals were collected at the European Synchrotron Radiation Facility (ESRF). As previously reported [28], data to 1.38 Å resolution were collected on beamline ID14-2 from a crystal cryo-cooled in 80% saturated Li2SO4 as a cryoprotectant. Data for MT7-1/1 were collected on beamline ID14-4 at 100°K from single crystal to 1.3 Å resolution. The crystal was found to belong to the space group P212121 with cell parameters 27.1 Å, 72 Å, 89.4 Å and three molecules in the asymmetric unit (Table S1). For MT7-1/3 data extending to 1.39 Å resolution were collected on beamline ID29. The crystals also belong to the space group P212121 but with different cell parameters: 25.8 Å, 56.6 Å, 80.7 Å; with two molecules in the asymmetric unit. The data were processed using MOSFLM [41] and reduced using programs from the CCP4 suite of programs. Molecular replacement was carried out with MOLREP [42] using the diiodo-MT7 (PDB code = 2VLW) as the starting model. After restrained refinement using REFMAC [43] the amino acid differences between the two loops were corrected according to the sequence. Density fitting and refinement were carried out with the aid of electron density maps (omit σA-weighted 2Fo- Fc and Fo-Fc) calculated and displayed using the XtalView [44] suite of programs and COOT [33]. Stereochemical analysis of the final refined model was checked with the validation tools in COOT. Waters were checked with phenix.refine [45]. Refinement statistics are detailed in Table S1 (MT1 R  = 19.3% Rfree = 23.4%); (MT7-1/1 R  = 21.4% Rfree  = 23.3%); (MT7-1/3 R  = 21.2% Rfree = 24.9%). The figures were made with PYMOL from DeLano Scientific LLC (The pyMOL Molecular Graphics System. DeLano Scientific, San Carlos, CA, USA).

### Protein Data Bank Accession Numbers

The coordinates and structure factors for the chimeric toxins have been deposited in the RCSB Protein Data Bank with the following ID codes: MT1∶4DO8; MT7-1/1∶3FEV; MT7-1/3∶3NEQ.

### CHO Cells and Membrane Preparation

Profs. P. O. Couraud & A. D. Strosberg (ICGM, Paris, France) kindly provided CHO cells stably expressing the cloned human muscarinic M_1_ and M_4_ receptors. The cells were grown in plastic Petri dishes (Falcon) which were incubated at 37°C in an atmosphere of 5% CO_2_ and 95% humidified air in Ham F12 medium pre-complemented with L-glutamine and bicarbonate (Sigma) supplemented with 10% fœtal calf serum and 1% penicillin/streptomycin (Sigma)**.** At 100% confluence, the medium was removed and the cells were harvested using Versen buffer (PBS +5 mM EDTA). They were washed with ice-cold phosphate buffer and centrifuged at 1700 g for 10 min (4°C). The pellet was suspended in ice-cold buffer (1 mM EDTA, 25 mM Na phosphate, 5 mM MgCl_2_, pH 7.4) and homogenized using an Elvehjem-Potter homogenizer (Fisher Scientific Labosi, Elancourt, France). The homogenate was centrifuged at 1700 g for 15 min (4°C). The sediment was resuspended in buffer, homogenized and centrifuged at 1700 g for 15 min (4°C). The combined supernatants were centrifuged at 35000 g for 30 min (4°C) and the pellet was suspended in the same buffer (0.1 ml/dish). Protein concentrations were determined according to the Lowry method using bovine serum albumin as standard. The membrane preparations were aliquoted and stored at –80°C. α_1A_-adrenoceptor cDNA inserted into the prK5 vector was kindly provided by Michael Brownstein (Craig Venter Institute, Rockville, MD). COS cells grown at 37°C under 5% CO_2_ in Dulbecco’s modified Eagle’s medium containing 10% fetal calf serum, 1% penicillin and 1% glutamine (Sigma-Aldrich, St Quentin-Fallavier, France). At 80% confluence, cells were transfected using calcium phosphate precipitation to transiently express the α_1A_-adrenoceptor. After 48h incubation at 37°C, cells were harvested and the membranes were prepared as previously described [27].

### Equilibrium [^3^H]-NMS and [^3^H]-Prazosin Binding Assays

The effect of MT7, MT1 toxins and various chimeric toxins on the equilibrium binding of [^3^H]-NMS for muscarinic M_1_ and M4 receptors and [^3^H]-Prazosin for α1a-adrenoceptor were determined with equilibrium inhibition binding experiments. For [^3^H]-NMS binding, membrane protein concentrations, adjusted so no more than 10% of added radio-ligand was specifically bound (around 1500–2000 cpm), were incubated in PBS-BSA at 25°C for 18 to 22 h, with varying concentrations of toxin and [^3^H]-NMS (0.5 nM), in a final assay volume of 300 µl. Non-specific binding was determined in the presence of 50 µM atropine. The reaction was stopped by addition of 3 ml of ice-cold buffer (PBS) immediately followed by filtration through Whatman GF/C glass fiber filters pre-soaked in 0.5% polyethylenimine. The filters were washed once again with 3 ml ice-cold buffer (PBS), dried and the bound radioactivity was counted by liquid scintillation spectrometry. Binding experiments with [^3^H]-Prazosin (1.5 nM) were performed in a 100 µL reaction mix at room temperature in buffer composed of 50 mM Tris-HCl, pH 7.4, 10 mM MgCl_2_, 1 g/L BSA. Incubation was stopped by filtration through 96 GF/C filter plates pre-incubated with 0.5% polyethylenimine. 25 µL of Microscint 0 were added onto each dry filter and the radioactivity was quantified on a TopCount beta counter (PerkinElmer, Courtaboeuf, France). Non specific binding were measured in presence of prazosin (10 µM). Each experiment was done in duplicate at least three times.

### Data Analysis

The binding data from individual experiments (n≥3) were analyzed by nonlinear regression analysis using Kaleidagraph 4.0 (Synergy Software, Reading, PA). After subtraction of the non-specific binding and normalization, data obtained with [^3^H]-NMS and [^3^H]-prazosin were analyzed using the Hill equation to estimate the IC_50_ and the slope factor, *n*_H_, of the inhibition curve. The affinities of toxins in inhibiting the binding of radiotracers, expressed as p*K*_i,_ were calculated from the IC_50_ values by applying the Cheng-Prusoff correction. Following the results obtained on the[^3^H]-NMS dissociation experiments on M1 receptor, highlighting the allosteric behaviour of MT7, MT7-1/1and MT7-1/3 toxins, the allosteric ternary complex model [46] was used to analyze the data on the effect of these toxins on the specific binding of the orthosteric radioligand [3H]-NMS.

## Supporting Information

Figure S1 **Overall far-UV CD spectra pattern of the different toxins and chimera.** The CD spectra were monitored in water, at 20°C with a peptide concentration of 10 µM.
(PDF)

Figure S2 **Schematic representation of MT7 (green), MT1 (gold) and chimeras MT7-1/1 (magenta) and MT7-1/3 (cyan) superimposed on each other on loop 2. a)** The superimposition of MT1 onto MT7 shows important deviation in both loops and in the sequence conserved disulphide-bridged scaffold but good conservation of the conformation of loop 2 and its interaction with the second strand of loop 3. This interaction is conserved in the chimeras. **b)** Chimera MT7-1/1 superimposes well on MT7 except for the grafted loop 1 which adopts the conformation and orientation seen in MT1. **c)** Chimera MT7-1/3 superimposes well on MT1 except for loop 1 which maintains the conformation and orientation seen in MT7. **d)** Chimera MT7-1/3 superimposes poorly on MT7. It maintains the interaction between loops 1 and 2 and but this implies that the disulphide-bridged scaffold adopts an orientation rotated with respect to the first two loops.
(PDF)

Figure S3 **Space-filling representation of MT7 and MT1.** The residues from loop 1 colored in green (conserved in lighter shade), residues from loop 2 in blue (tip) and cyan (top) with the conserved residues in lighter shades, loop 3 in yellow (conserved in lighter shade) and the C-terminal residue in magenta. **a)** Front view of MT7 showing the interacting residues from the three loops. **b)** Back view of MT7 showing the interacting residues from the three loops and the C-terminal residue. **c)** Front view of MT7 with the residues from loop 2 top removed to show the lack of interactions between the other elements of the toxin assembly apart from the interaction of the C-terminal residue with loop 1. **d)** Front view of MT1 showing that the overall shape of the toxin differs substantially from that of MT7. **e)** Back view of MT1 showing variations in most elements, except the tip that maintains its orientation relative to the rest of the toxin as in MT7. f) With the top of loop 2 removed, MT1 shows that its C-terminal residue mediates an interaction between loops 1 and 3 and that the tip of loop 2 is stabilized by the longer loop 3.
(PDF)

Table S1 **Crystallization conditions and data collection statistics.**
(DOCX)
