# Supplementary material for: Engineering of Three-Finger Fold Toxins Creates Ligands with Original Pharmacological Profiles for Muscarinic and Adrenergic Receptors
Source: PLoS One. 2012 Jun 14;7(6):e39166. doi: 10.1371/journal.pone.0039166 (PMC3375269; doi:10.1371/journal.pone.0039166)
Supplement: Figure S1 — Overall far-UV CD spectra pattern of the different toxins and chimera. The CD spectra were monitored in water, at 20°C with a peptide concentration of 10 µM. (PDF) [file pone.0039166.s001.pdf]

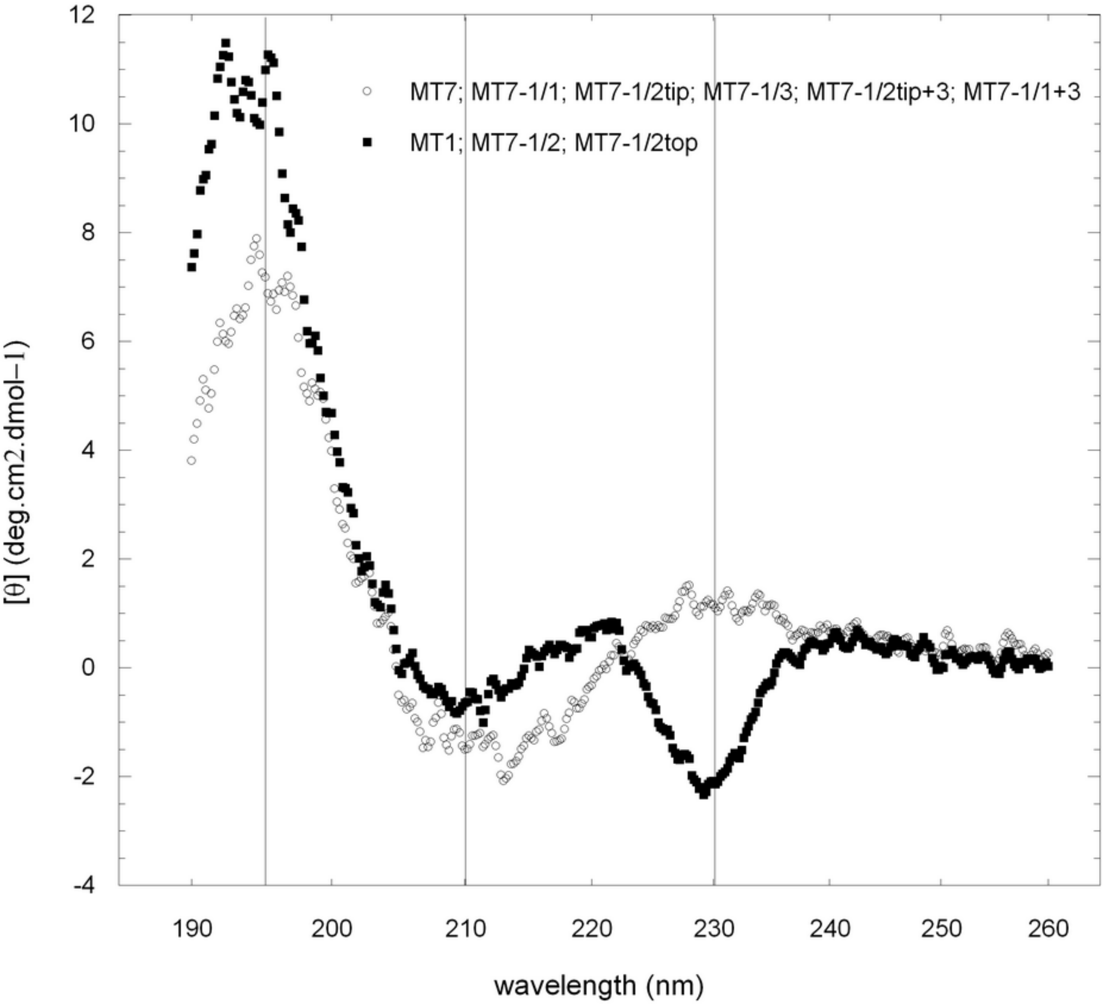

Figure S1. **Overall far-UV CD spectra pattern of the different toxins and chimera**. The CD spectra were monitored in water, at 20 C° with a peptide concentration of 10 µM.
