# Supplementary material for: Engineering of Three-Finger Fold Toxins Creates Ligands with Original Pharmacological Profiles for Muscarinic and Adrenergic Receptors
Source: PLoS One. 2012 Jun 14;7(6):e39166. doi: 10.1371/journal.pone.0039166 (PMC3375269; doi:10.1371/journal.pone.0039166)
Supplement: Figure S2 — Schematic representation of MT7 (green), MT1 (gold) and chimeras MT7-1/1 (magenta) and MT7-1/3 (cyan) superimposed on each other on loop 2. a) The superimposition of MT1 onto MT7 shows important deviation in both loops and in the sequence conserved disulphide-bridged scaffold but good conservation of the conformation of loop 2 and its interaction with the second strand of loop 3. This interaction is conserved in the chimeras. b) Chimera MT7-1/1 superimposes well on MT7 except for the grafted loop 1 which adopts the conformation and orientation seen in MT1. c) Chimera MT7-1/3 superimposes well on MT1 except for loop 1 which maintains the conformation and orientation seen in MT7. d) Chimera MT7-1/3 superimposes poorly on MT7. It maintains the interaction between loops 1 and 2 and but this implies that the disulphide-bridged scaffold adopts an orientation rotated with respect to the first two loops. (PDF) [file pone.0039166.s002.pdf]

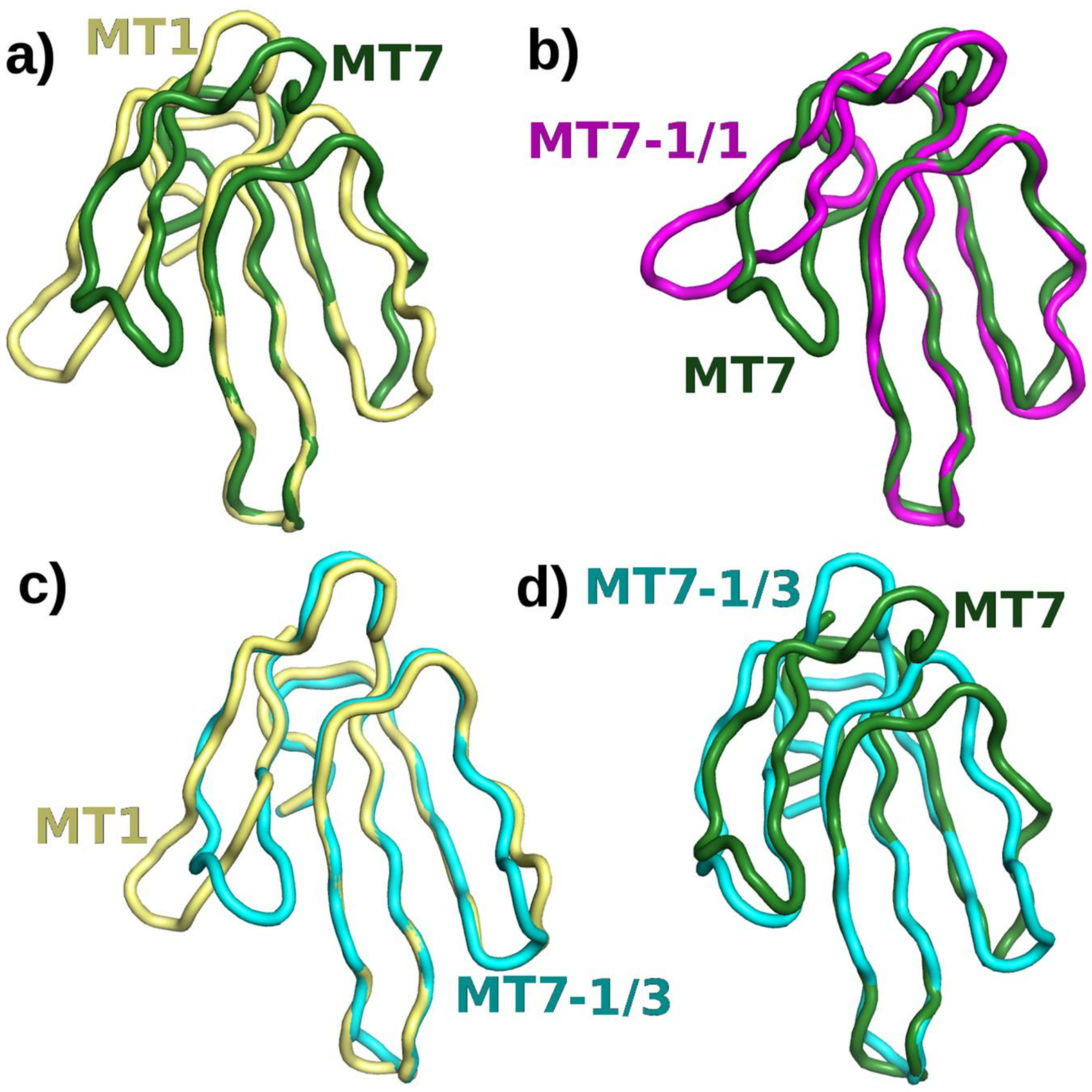

Figure S2. Schematic representation of MT7 (green), MT1 (gold) and chimeras MT7-1/1 (magenta) and MT7-1/3 (cyan) superimposed on each other on loop 2.

a) The superimposition of MT1 onto MT7 shows important deviation in both loops and in the sequence conserved disulphide-bridged scaffold but good conservation of the conformation of loop 2 and its interaction with the second strand of loop 3. This interaction is conserved in the chimeras. **b)** Chimera MT7-1/1 superimposes well on MT7 except for the grafted loop 1 which adopts the conformation and orientation seen in MT1. **c)** Chimera MT7-1/3 superimposes well on MT1 except for loop 1 which maintains the conformation and orientation seen in MT7. **d)** Chimera MT7-1/3 superimposes poorly on MT7. It maintains the interaction between loops 1 and 2 and but this implies that the disulphide-bridged scaffold adopts an orientation rotated with respect to the first two loops.
