# Supplementary material for: Engineering of Three-Finger Fold Toxins Creates Ligands with Original Pharmacological Profiles for Muscarinic and Adrenergic Receptors
Source: PLoS One. 2012 Jun 14;7(6):e39166. doi: 10.1371/journal.pone.0039166 (PMC3375269; doi:10.1371/journal.pone.0039166)
Supplement: Figure S3 — Space-filling representation of MT7 and MT1. The residues from loop 1 colored in green (conserved in lighter shade), residues from loop 2 in blue (tip) and cyan (top) with the conserved residues in lighter shades, loop 3 in yellow (conserved in lighter shade) and the C-terminal residue in magenta. a) Front view of MT7 showing the interacting residues from the three loops. b) Back view of MT7 showing the interacting residues from the three loops and the C-terminal residue. c) Front view of MT7 with the residues from loop 2 top removed to show the lack of interactions between the other elements of the toxin assembly apart from the interaction of the C-terminal residue with loop 1. d) Front view of MT1 showing that the overall shape of the toxin differs substantially from that of MT7. e) Back view of MT1 showing variations in most elements, except the tip that maintains its orientation relative to the rest of the toxin as in MT7. f) With the top of loop 2 removed, MT1 shows that its C-terminal residue mediates an interaction between loops 1 and 3 and that the tip of loop 2 is stabilized by the longer loop 3. (PDF) [file pone.0039166.s003.pdf]

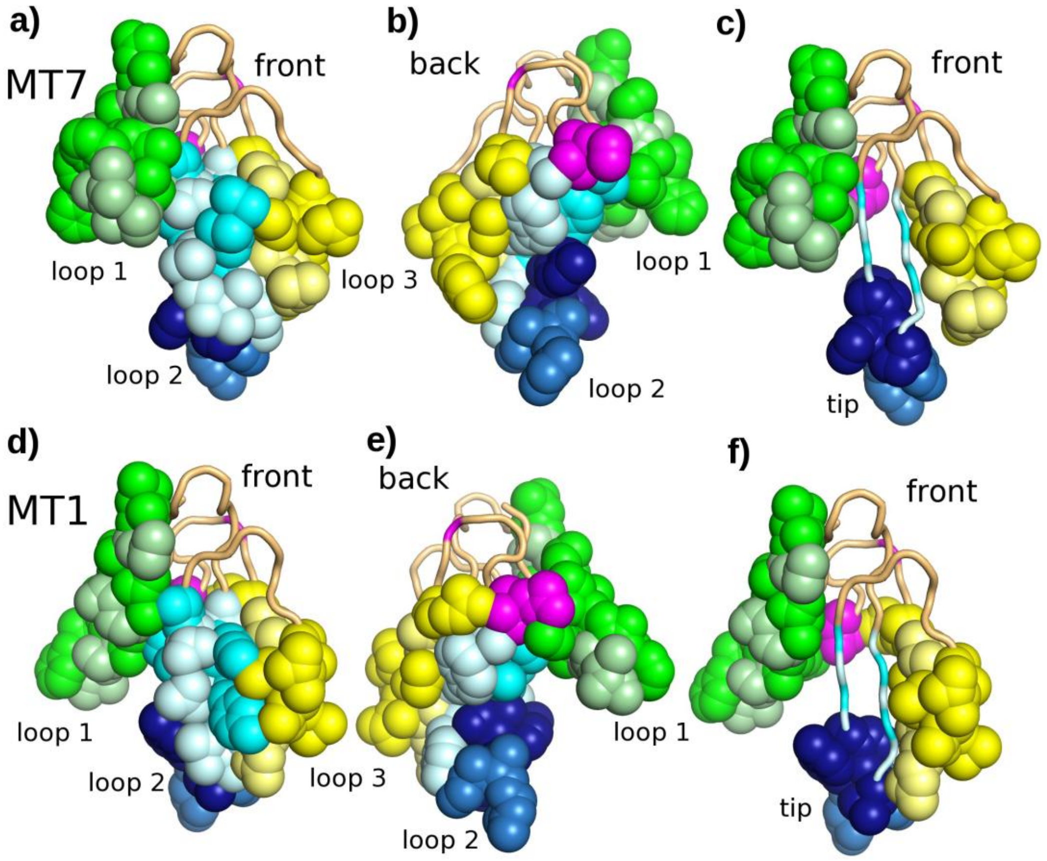

Figure S3. **Space-filling representation of MT7 and MT1**. The residues from loop 1 colored in green (conserved in lighter shade), residues from loop 2 in blue (tip) and cyan (top) with the conserved residues in lighter shades, loop 3 in yellow (conserved in lighter shade) and the C-terminal residue in magenta. **a)** Front view of MT7 showing the interacting residues from the three loops. **b)** Back view of MT7 showing the interacting residues from the three loops and the C-terminal residue. **c)** Front view of MT7 with the residues from loop 2 top removed to show the lack of interactions between the other elements of the toxin assembly apart from the interaction of the C-terminal residue with loop 1. **d)** Front view of MT1 showing that the overall shape of the toxin differs substantially from that of MT7. **e)** Back view of MT1 showing variations in most elements, except the tip that maintains its orientation relative to the rest of the toxin as in MT7. f) With the top of loop 2 removed, MT1 shows that its C-terminal residue mediates an interaction between loops 1 and 3 and that the tip of loop 2 is stabilized by the longer loop 3.
