# Supplementary material for: Engineering of Three-Finger Fold Toxins Creates Ligands with Original Pharmacological Profiles for Muscarinic and Adrenergic Receptors
Source: PLoS One. 2012 Jun 14;7(6):e39166. doi: 10.1371/journal.pone.0039166 (PMC3375269; doi:10.1371/journal.pone.0039166)
Supplement: Table S1 — Crystallization conditions and data collection statistics. (DOCX) [file pone.0039166.s004.docx]

**Table S1: Crystallization conditions and data collection statistics**

| **CRYSTALLIZATION:** Toxins lyophilized and re-dissolved in 50 mM sodium acetate. | | | |
| --- | --- | --- | --- |
| **MT1** | ***Protein:*** 8.3 mg/ml in 0.02% NaN_3_, 0.05M sodium acetate, pH 5.5.  ***Precipitant:*** 36% MPEG 2K 0.1 mM imidazole-HCl, 0.45 M NaCl, 0.09 M KSCN by sitting drop vapor diffusion. No cryoprotectant needed. | | |
| **MT7-1/1** | ***Protein:*** 5 mg/ml in 0.02% NaN_3_, 0.05 M sodium acetate pH 5.5.  ***Precipitant:*** 1M ammonium sulfate, 90 mM sodium citrate, 8% 1,6 hexanediol, 6% propanol, pH 6.0 by sitting drop vapor diffusion.  ***Cryoprotectant:*** 80% saturated Li_2_SO_4_. | | |
| **MT7-1/3** | ***Initial conditions:*** 5 mg/mL in 0.02% NaN_3_, 0.05M sodium acetate pH 5.5. Crystallization by slow evaporation without precipitant.  ***Cryoprotectant:*** 80% saturated Li_2_SO_4_. | | |
| **CRYSTALLOGRAPHY** | | | |
| ***Toxins***  *PDB code* | **MT1**  *4DO8* | **MT7-1/1**  *3FEV* | **MT7-1/3**  *3NEQ* |
| **DATA COLLECTION** | ESRF-ID23-1 | ESRF-ID14-1 | ESRF-ID29 |
| Space group | C2 | P 2_1_ 2_1_ 2_1_ | P 2_1_ 2_1_ 2_1_ |
| cell parameters (Å) | 32.3 116.0 46.2  β=108° | 27.14 71.97 89.42 | 25.8 56.6 80.7 |
| Number mol. / asym. u. | 2 | 3 | 2 |
| Resolution range | 50-1.8 Å | 28-1.3 Å | 46-1.25 Å |
| Reflections Observed | 14793 | 39130 | 31850 |
| Completion | 98.9% | 88.5% | 99.8% |
| R_Sym_^a^ | 8.1% | 6.5% | 8.8% |
| Redundancy | 4.1 | 5.5 | 7.5 |
| **STRUCTURE** | | | |
| Resolution | 30-1.8 Å | 28-1.3 Å | 46-1.25 Å |
| Number of Reflections | 14044 | 38919 | 31850 |
| R_work_^b^ / R_free_^c^ (%) | 19.3 / 23.4 | 21.5 / 23.3 | 14.9 / 18.7 |
| Favored regions | 99% | 98% | 96.3% |
| Allowed | >99.9% | >99.9% | >99.9% |
| Outliers | none | none | none |

^a^R_sym_ = ∑_hkl_∑_i_|I_i_–‹I›|∑_hkl_∑_i_ I_i_

^b^R_work_ = ∑_hkl_||F_obs_|–k|F_calc_||/∑_hkl_|F_obs_|,

^c^R_free_ was calculated using 5% of data excluded from refinement.
